# Late thrombosis of a Transcatheter aortic valve: the border between a proactive and reactive management

**DOI:** 10.1186/s13019-018-0816-4

**Published:** 2018-12-17

**Authors:** Marco Gennari, Gianluca Polvani, Mauro Pepi, Francesco Arlati, Andrea Annoni, Marco Agrifoglio

**Affiliations:** 1 Centro Cardiologico Monzino, IRCCS, Via Parea 4, 20138 Milan, Italy; 2 Department of Cardiovascular Sciences and Community Health, University of Milan, Milan, Italy

**Keywords:** Transcatheter aortic valve replacement, Valve thrombosis, ECMO

## Abstract

**Background:**

Valve thrombosis – either biological or mechanical – is proved to increase patient’s morbidity and mortality. No consensus exist on the best management in such cases.

**Case presentation:**

We report the case of a 69-year-old man presenting with a late thrombosis of a transcatheter aortic valve who was medically managed until he acutely worsened, developing myocardial ischemia and cardiogenic shock.

**Conclusion:**

This unlucky case raises a word of caution about the safety of a reactive management.

## Background

Prosthetic valve thrombosis is known to carry relevant morbidity and mortality. Little is known about its occurrence after transcatheter aortic valve replacement (TAVR); all the information we have are derived from case reports and relatively small series of patients [1].

The current postoperative management suggests dual antiplatelet therapy (DAPT) to reduce the risk of valve thrombosis, even if more and more evidences reveal the potential superiority of oral anticoagulation [2] A more serious issue regards the appropriate management once the valve thrombosis has occurred.

## Case presentation

A 69-year-old man was referred to our Emergency Department for resting dyspnea after 2 months progressive shortness of breath, 2 years after transcatheter aortic valve replacement (TAVR).

In 1994 he underwent aortic valve replacement with a 23 mm Biocor™ valve (*St. Jude, MN, USA*) for native valve endocarditis. Eleven years later (2015) he was re-operated for structural valve deterioration. In that occasion – due to the presence of extreme calcification of the aortic annulus and root – we had to replace the prosthesis with a 23 mm Edwards Sapien 3 transcatheter valve (*Edwards Lifesciences, Irvine, CA*) under direct view, as previously described [3].

The patient revealed anticoagulants discontinuation due to excessive bleeding after surgery for a chordoma of the nose within the last 2 months (he was on anticoagulant after an episode of atrial fibrillation). He took Aspirin 100 mg/day with a good compliance. He had no history of hyper-coagulation state or previous documented thrombosis.

**Fig. 1:**
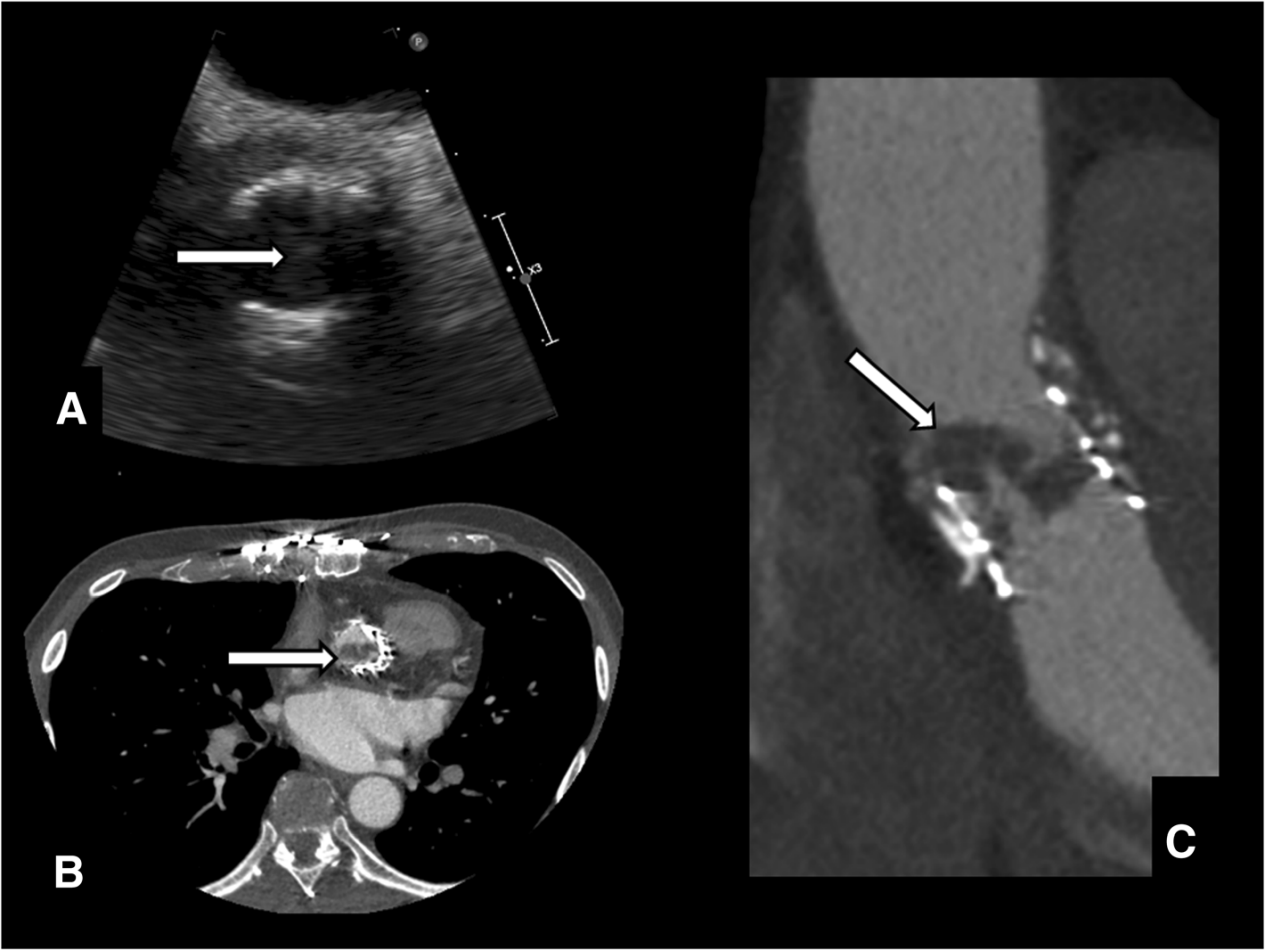
**a** The echocardiogram was suggestive for valve thrombosis (*arrow*). **b & c.** The finding was confirmed on thoracic computed tomography (*arrows*)

The transthoracic echocardiogram showed increased trans-valvular gradients (mean left ventricular outflow tract/aorta gradient of 62 mmHg with 0,43cm^2^ of valvular area) and ipo-echogenic images evocative of intra-valvular thrombosis. A thoracic computed tomography (CT) confirmed the presence of valvular thrombosis (Fig. 1) in the presence of diseased-free coronary anatomy. After discussion in the Heart Team setting it was decided to attempt systemic anticoagulation with heparin to achieve dissolution of the thrombus. After 1 week of systemic anticoagulation a control CT did not show any evidence of improvement, so we planned a surgical re-intervention. The day before the scheduled surgery the patient suddenly experienced thoracic pain and electrocardiographic signs of myocardial ischemia. An urgent coronary angiography was performed while the clinical and hemodynamic state worsened. He had a cardiac arrest during the procedure; an immediate cardio-pulmonary resuscitation (CPR) was performed while a peripheral extracorporeal membrane oxygenation (ECMO) support was instituted and the patient transferred into the operating room for an emergent surgery.

**Fig. 2:**
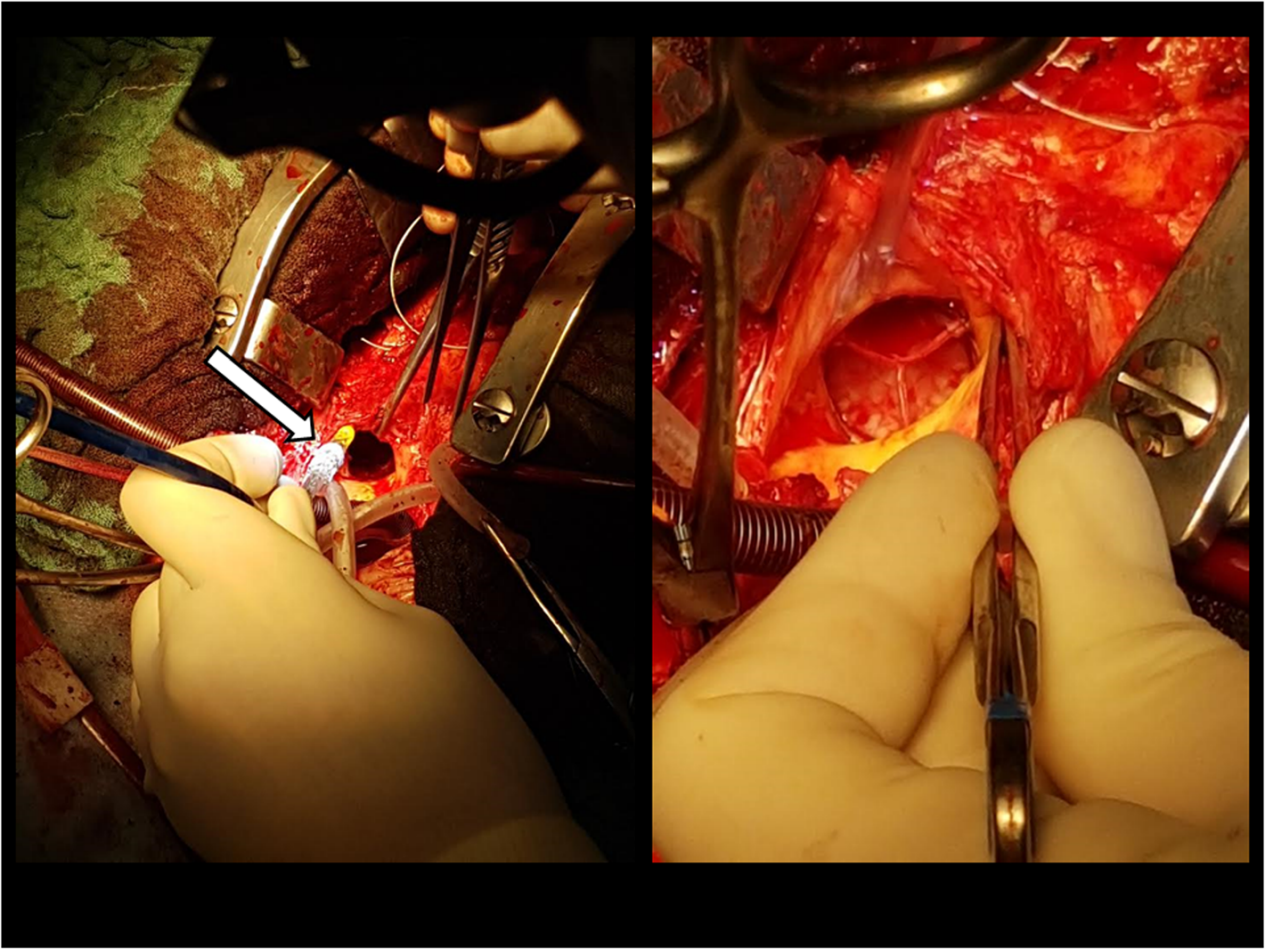
Intraoperative images. The new transcatheter valve deployed under direct view (*arrow*)

**Fig. 3:**
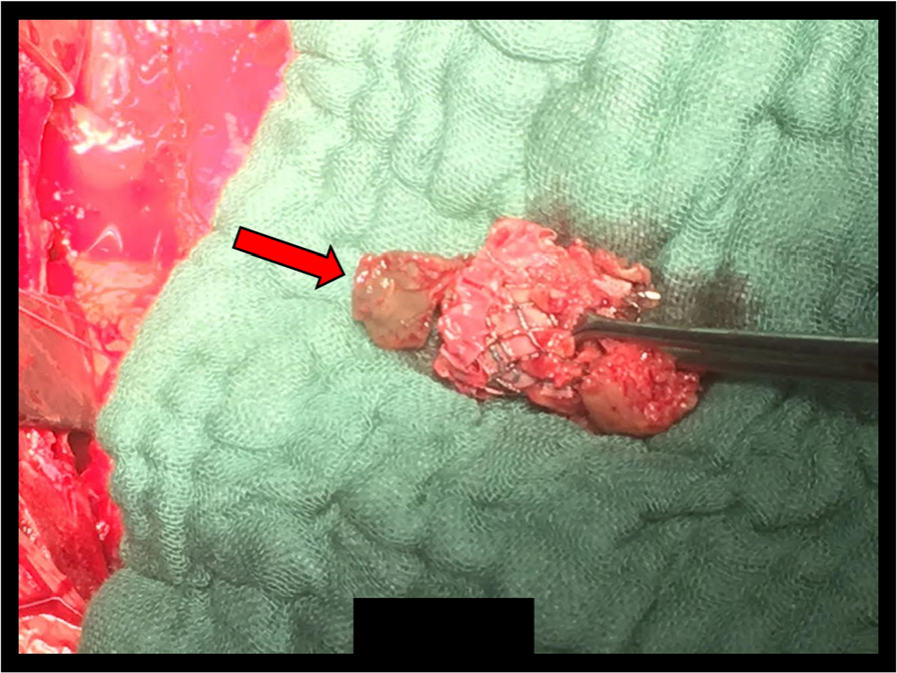
Intraoperative image of the excised transcatheter valve with the adherent thrombus (*arrow*)

After cardioplegic cardiac arrest and transverse aortotomy the thrombosed transcatheter valve was excised and a new 23 mm Edwards Sapien 3 valve was deployed in an off-label fashion under direct view with good echocardiographic result (Fig. 2). The valve thrombosis was confirmed once excised (Fig. 3).

Unfortunately he developed an incoercible cardiogenic and septic shock and finally died on the 10th postoperative day.

## Discussion

Prosthetic valve thrombosis (PVT) accounts for any thrombus formation attached to or near a prosthetic valve that is not caused by infection and determining some degree of valve obstruction and dysfunction and that carries significant morbidity and mortality to the patient.

Some degree of reduced leaflet motion is a detected in 10 to 15% of TAVR patients and generally resolves after anticoagulation therapy. [4] Although currently either the North American and European Society of Cardiology suggest DAPT following TAVR procedures, more and more clinical evidences seem to propose a more appropriate management by oral anticoagulants after transcatheter aortic valve replacement.

No consensus exists regarding the best treatment option once the thrombus has developed.

In this case the biggest challenge was balancing a potential life–threatening condition caused by valve thrombosis and its initial dysfunction and the extremely elevated surgical risk due to the third cardiac operation after surgical implantation of a transcatheter valve due to porcelain aortic root and annulus. Thus, the first-line we choose was trying to manage the patient conservatively with systemic heparinization and close clinical and instrumental monitoring.

In this case the localized aggressive tumor and the relevant postoperative bleeding may have impaired the hemostatic status of the patient, shifting it toward a pro-coagulant one.

We speculate that the transcatheter valve forced against the stiff aortic ring of the previous transcatheter valve and the rigidity and narrowing of the degenerated homograft has caused a progressive thrombosis occurred within the stented portion of the valve towards the leaflets once the hemostasis perturbation occurred leading to the catastrophic consequence.

## Conclusion

From this unlucky case a word of caution arises about the medical management of such TAVR population; although a single case report does not provide wider conclusion we believe that a very close monitoring of the clinical ad morphologic valve features are mandatory while the surgical option still remains valid once an early evidence of no improvement after adequate anticoagulation therapy.

## Abbreviations

CPR: Cardio-pulmonary resuscitation
DAPT: Dual antiplatelet therapy
ECMO: Extracorporeal membrane oxygenation
NOACs: The novel anti coagulants
PVT: Prosthetic valve thrombosis
TAVR: Transcatheter aortic valve replacement
